# From Isolation to Collaboration: Data Trading Mechanism in the Era of Large Language Model Democratization

**DOI:** 10.34133/research.1336

**Published:** 2026-06-26

**Authors:** Kang Wang, Hongxun Hui

**Affiliations:** ^1^ State Key Laboratory of Internet of Things for Smart City, University of Macau, Macao, China.; ^2^ Department of Electrical and Computer Engineering, University of Macau, Macao, China.

## Abstract

Extensive data resources are critical for broadening the application scope and improving the performance of large language models (LLMs). Local deployment of LLMs enables users to leverage the advanced capabilities of LLMs while maintaining secure access to local data. Therefore, local LLMs deliver more domain-specific services than general LLMs. However, concerns over data security and the absence of economic incentives restrict cross-industry collaborative utilization of data resources. To address this challenge, an interdisciplinary framework integrating artificial intelligence, economic theory, and data security technologies is required to explore the data trading mechanism for local LLMs. The key aspects of a data market framework, economic evaluation for data pricing, and trusted data trading are analyzed to support efficient utilization of cross-industry data resources. By constructing a data trading mechanism, industry-specific local LLMs can be further enhanced through cross-industry knowledge integration, thereby improving productivity across sectors.

## Democratization of LLMs and Emerging Need for Data Trading

**Fig. 1.**
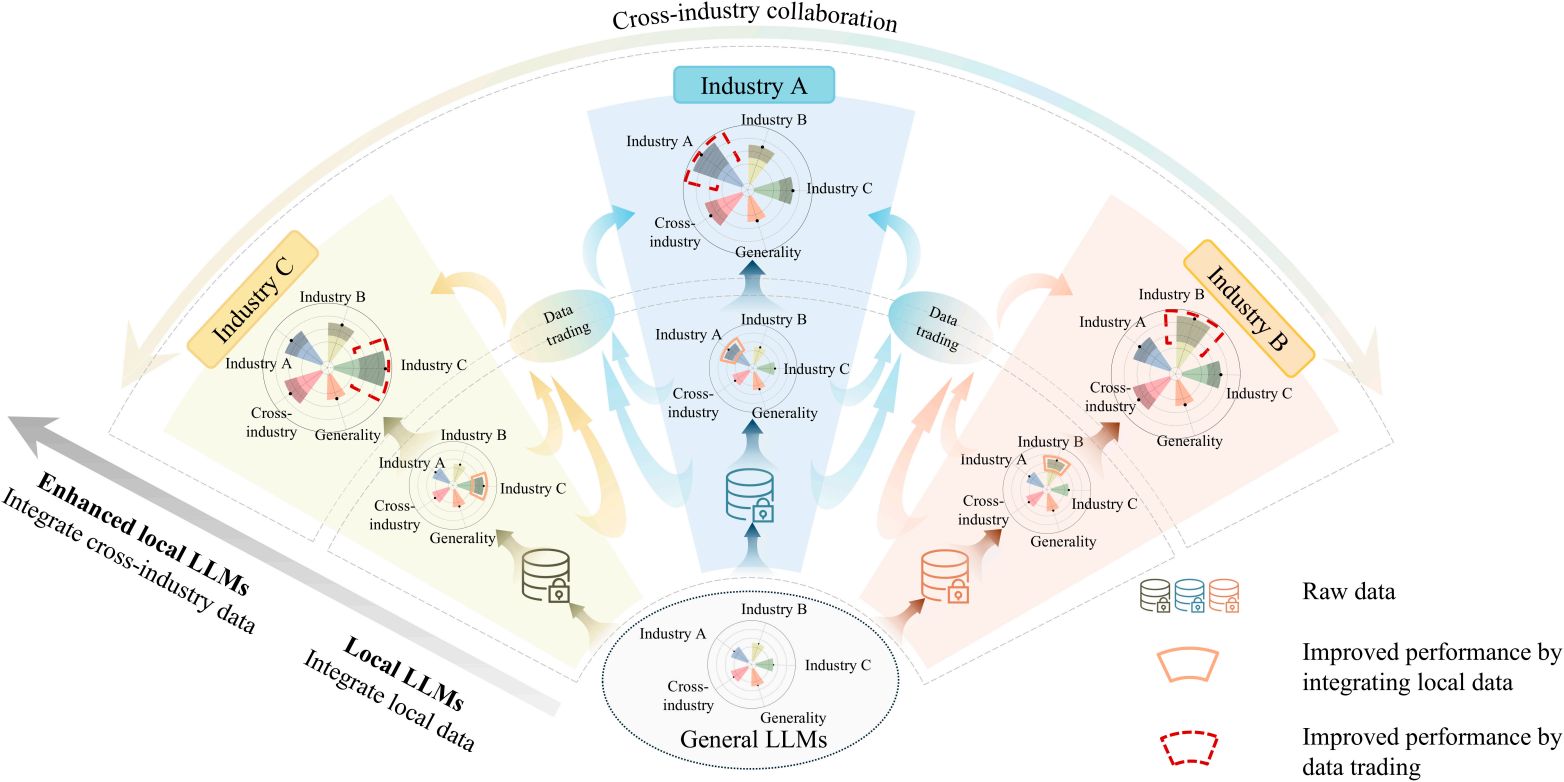
Data resources have an impact on the performance of local large language models (LLMs). From the perspective of a given industry, incorporating local data into general LLMs produces industry-specific local LLMs and strengthens LLMs’ capabilities in this industry. Leveraging data resources from other industries further produces enhanced local LLMs and substantially improves performance in this industry. From the perspective of cross-industry, trading data resources across industries facilitates knowledge integration.

Local large language models (LLMs), deployed within an organization with secure access to local data [1], allow users to leverage internal data for domain-specific tasks while maintaining privacy [2]. This increased accessibility marks a critical step toward the democratization of LLMs [3]. Compared with general LLMs, industry-specific local LLMs have the potential to perform better by leveraging local data and cross-industry data resources (Fig. 1). Such cross-industry data resources may further enhance local LLMs by improving model capability, cross-domain adaptability, and downstream task performance [4].

Local LLMs facilitate the utilization of local data within individual organizations. However, cross-industry data sharing remains constrained by privacy risks and limited mutual trust. This indicates that data democratization remains incomplete. To effectively capitalize on local LLMs, industries need not only leverage their own local data but also gain access to relevant external data resources from other industries [5]. Therefore, the data trading mechanism is essential for unlocking cross-industry knowledge integration. This paper discusses cross-industry data resource trading for local LLMs from the perspectives of trading framework, economic evaluation, and trusted trading.

## Cross-Industry Data Trading

Data trading among industry-specific local LLMs can be divided into data product trading and raw data trading [6]. The former exchanges analytical information to support decision-making [7], while the latter exchanges data for model training [8]. Data product trading and raw data trading differ in their tradable objects, processing burden, application scenarios, and governance requirements. The data trading mechanism provides an effective pathway for cross-industry data sharing and benefit allocation among local LLMs.

## Core Components of Data Trading among Local LLMs

An effective data resource trading mechanism for local LLMs rests on the following 3 foundational pillars.

The data trading framework provides a platform for the efficient circulation of heterogeneous data. Data product trading relies on access-based delivery and logging, while raw data trading relies on data delivery with access boundaries. Data product trading and raw data trading possess distinctive characteristics in delivery frequency, transmission requirements, processing costs, and computational burdens. The market needs an adaptive trading mechanism to handle the heterogeneous characteristics. Intelligent scheduling and resource orchestration can maintain smooth data flows under dynamic workloads. Data standardization and format conversion services can reduce processing costs for local LLM users.

Economic evaluation provides the basis for data valuation. The economic value of data is reflected in application-specific benefits. Therefore, an application-based paradigm is essential for data valuation and benefit allocation. For example, leave-one-out analysis can be utilized to estimate the marginal contribution. Sensitivity analysis can be utilized to examine robustness under changes in data quality, volume, and delivery frequency.

Trusted trading provides the basis for secure execution of data trading. Both data product trading and raw data trading face risks related to malicious manipulation, unauthorized reuse, and data or model compromise. Therefore, effective safeguards are required to preserve confidentiality, integrity, and controllability. Representative approaches include application programming interface-based access, encryption, federated learning, blockchain, and related technologies.

## Conclusion

The data utilization paradigm has been fundamentally transformed by the democratization of LLMs. To enhance the performance of local LLMs, industries should not only leverage local data but also foster cross-industry collaboration through data trading. By integrating interdisciplinary solutions, the data trading mechanism can be a pivotal enabler for efficient circulation of diverse data resources. Advances in data trading framework, economic evaluation, and trusted trading will be critical for facilitating cross-industry collaboration and unlocking the potential of LLMs.
